# Forty-six cases of nasopharyngeal carcinoma treated with 50 Gy radiotherapy plus hematoporphyrin derivative: 20 years of follow-up and outcomes from the Sun Yat-sen University Cancer Center

**DOI:** 10.1186/s40880-016-0098-y

**Published:** 2016-04-07

**Authors:** Bing-Qing Xu, Zi-Wei Tu, Ya-Lan Tao, Zhi-Gang Liu, Xiao-Hui Li, Wei Yi, Chang-Bing Jiang, Yun-Fei Xia

**Affiliations:** State Key Laboratory of Oncology in South China, Department of Radiation Oncology, Collaborative Innovation Center for Cancer Medicine, Sun Yat-sen University Cancer Center, 651 Dongfeng East Road, Guangzhou, 510060 Guangdong P.R. China; Department of Radiation Oncology, The First Affiliated Hospital of Guangzhou Medical University, Guangzhou, 510060 Guangdong P.R. China

**Keywords:** Low-dose radiotherapy, Nasopharyngeal carcinoma, Radiosensitivity, Quality of life

## Abstract

**Background:**

With the improved overall survival (OS) of nasopharyngeal carcinoma (NPC) patients, the importance of quality of life (QoL) is increasingly being recognized. For some radiosensitive NPC patients, whether low-dose radiotherapy can improve the QoL without affecting clinical efficacy is unknown. This study aimed to assess the survival rates and QoL of NPC patients treated with 50 Gy radiotherapy plus hematoporphyrin derivative (HPD).

**Methods:**

Forty-six newly diagnosed NPC patients treated with 50 Gy radiotherapy plus HPD between June 1988 and July 1992 were analyzed. All patients were restaged according to the 7th edition of the American Joint Committee on Cancer staging system. The radiotherapy plan was designed on the basis of pretreatment computed tomography. The OS, local recurrence-free survival (LRFS), distant metastasis-free survival (DMFS), and disease-free survival (DFS) rates were estimated using the Kaplan–Meier method. QoL was assessed using the Late Radiation Morbidity Scoring Criteria of the Radiation Therapy Oncology Group.

**Results:**

The 5-year OS, LRFS, DMFS, and DFS rates were 74.3%, 72.6%, 82.1%, and 61.2%, respectively. The corresponding 10-year rates were 38.4%, 62.9%, 78.5%, and 49.8%, respectively, and the 20-year rates were 27.7%, 51.4%, 78.5%, and 40.7%, respectively. None of the patients developed severe radiation-related complications, such as radiation-induced temporal lobe necrosis, hearing loss, trismus, and dysphagia.

**Conclusion:**

Some NPC patients were sensitive to 50 Gy radiotherapy plus HPD, and this sensitivity was characterized by long-term survival without significant late treatment morbidities.

## Background

Radiotherapy is a major treatment modality for nasopharyngeal carcinoma (NPC), and the 5-year survival rate is 66%–83% in NPC patients treated with radiotherapy alone [1–3]. The overall survival (OS) rates are greater than 90% for early-stage NPC patients [4, 5]. With the improved OS of NPC patients, the importance of quality of life (QoL) is increasingly being recognized. However, conventional definitive radiotherapy can lead to radiation-related complications [6–10], such as xerostomia, trismus, hearing loss, neck fibrosis, and radiation-induced temporal lobe necrosis, which affect the QoL of NPC patients. The current radiotherapy for NPC is a “catch-all” therapy, wherein all NPC patients are given a prescribed radiation dose of approximately 60–70 Gy, covering a heterogeneous group of tumors that range from radiosensitive to radioresistant. Thus, it is very important to improve the QoL of NPC patients without compromising the treatment efficacy and to provide individualized radiotherapy according to different radiosensitivities of NPCs. In 2006, we divided NPCs into the following four types: radiosensitive and non-metastasis-prone; radioresistant and non-metastasis-prone; radiosensitive and metastasis-prone; and radioresistant and metastasis-prone [11]. This typing provides a new approach for NPC radiation planning based on the radiosensitivity and metastatic tendency. However, this typing approach is limited because it does not consider other doses below or above the conventional radiotherapy doses.

In clinical practice, a 56-year-old man was diagnosed with undifferentiated NPC at clinical stage IV (T2N3M0) according to the Chinese 1992 staging system and at stage IVb (T1N3M0) according to the 7th American Joint Committee on Cancer (AJCC) staging system. His treatment plan involved conventional radical radiation therapy; however, he did not complete the planned treatment. As a result, the nasopharyngeal region was irradiated with a total dose of 36 Gy, and the irradiation dose to the neck lymph node drainage region was 30.1 Gy. Afterwards, the patient did not receive any treatment. However, he lived cancer-free for an additional 10 years [12]. This case led us to wonder what type of nasopharyngeal tumors can be controlled with low-dose radiotherapy and whether the case is generalizable. With these questions in mind, we retrospectively identified a series of NPC patients from the Sun Yat-sen University Cancer Center who were irradiated with only 50 Gy radiotherapy plus hematoporphyrin derivative (HPD) between June 1988 and July 1992 and then analyzed their 20-year survival rates and QoL.

HPD is a complex mixture of porphyrins prepared from hematoporphyrin-IX (HP). Typically, this mixture consists of 20% HP, 20%–30% hydroxy-ethyl-vinyl-deuteroporphyrin, and 3%–5% protoporphyrin. The other half is a mixture of dimers, trimers, and some higher molecular weight oligomers. Under the background of researches which were conducted 20 years ago, HPD was assumed to be a radiosensitizer and can enhance the sensitivity of some types of cancer cells to radiation [13, 14]. However, after more than 20 years of experience using HPD to treat tumors, recent studies showed that HPD was the most frequently used photosensitizer in photodynamic therapy (PDT) [15, 16]. This therapy is based on the administration of a photosensitizer followed by localized exposure of the target tissue to laser-generated light. Light is absorbed by the photosensitizer, which subsequently initiates the photo-chemical reaction to form cytotoxic agents within the irradiated field to kill neoplastic cells [17]. The photoexcitation of HPD generates singlet oxygen, which is suggested as the main damaging agent in PDT. HPD is a photosensitizer, not a radiosensitizer, and it works by laser rather than X-ray used in radiotherapy. Thus, HPD can’t enhance the radiosensitivity of nasopharyngeal cancer cells. Therefore, in this study, we don’t introduce too much about the role of HPD in the survival rates and QoL of NPC patients treated with low-dose radiotherapy plus HPD. This study analyzed the survival rates and QoL of NPC patients treated with 50 Gy radiotherapy plus HPD.

## Patients and methods

### Patients

The present study was on the basis of a clinical trial entitled “The optimal regimen of comprehensive treatment for nasopharyngeal carcinoma” (Grant No. 85-914-02) [18]. The patient inclusion criteria of that trial were as follows: (1) histological diagnosis of NPC; (2) no prior treatment before radiotherapy; (3) no distant metastases; and (4) Eastern Cooperative Oncology Group (ECOG)/World Health Organization (WHO)/Zubrod performance score less than four. The exclusion criteria were any of the following: (1) distant metastases; (2) presence of other malignancies; and (3) severe complications or active infection. The institutional review board approved the protocol, and all patients provided written informed consent for participation.

**Fig. 1:**
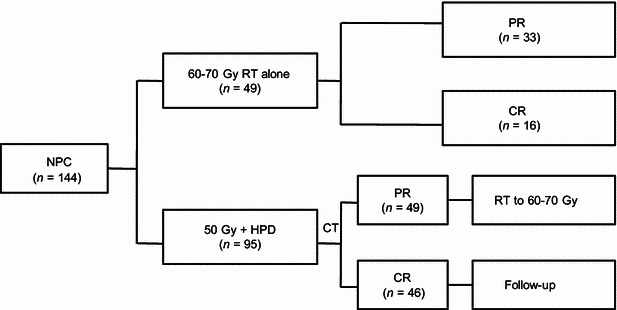
Flow chart of nasopharyngeal carcinoma (NPC) patient selection. *RT* radiotherapy; *HPD* hematoporphyrin derivatives; *CT* computed tomography; *PR* partial response; *CR* complete response

All patients underwent a pre-treatment evaluation that included a complete physical examination, fiberoptic nasopharyngoscopy, computed tomography (CT) scanning of the nasopharynx and neck, chest radiography, and abdominal sonography. The Chinese 1992 staging system [19] was used for stage classification. A total of 144 patients were enrolled in the previous study: 49 were given radiation treatment alone, and 95 were given HPD plus radiation treatment (Fig. 1). The patients in the latter group were divided into two subgroups according to their CT evaluation after receiving 50 Gy radiotherapy. If the CT evaluation showed a partial response, the primary tumor was irradiated with additional 10–20 Gy. If the CT evaluation showed a complete response, radiotherapy was stopped at 50 Gy. In the HPD plus radiation treatment group, 46 patients showed complete response after 50 Gy of radiation. Therefore, the 46 NPC patients were selected in this study and the 20-year follow-up data of them were analyzed in the present study. All patients were restaged according to the 7th edition of the AJCC staging system.

### Radiotherapy

All patients were given 50 Gy conventional external beam radiotherapy plus HPD. A daily fraction of 2 Gy and five fractions per week were delivered using a cobalt-60 or linear accelerator. Opposing lateral faciocervical fields were used in the two-dimensional conformal radiotherapy to cover the nasopharynx and upper cervical lymphatic drainage region, with one lower anterior cervical field to cover the lower cervical region. After 40 Gy, opposing lateral preauricular fields were used to cover the primary region and anterior split neck fields were used to cover the cervical region. The total dose to the primary tumor was 50 Gy, and the total dose to the lymph drainage region was 40–50 Gy. Treatment was completed within 5 weeks.

### Assessment of QoL

The QoL of NPC patients was assessed according to the functional assessment of cancer therapy-head and neck (FACT-H&N) V4 [20, 21] and the Radiation Therapy Oncology Group (RTOG) CTC3.0 radiation morbidity grading system [22]. These QoL measures included the following seven items: neck skin, hearing loss, dry mouth, brain injury, distance between two dens incisivus medialis, sleep, and appetite. Scores ranged from 0 to 4; a high score represented a relatively poor QoL.

### Follow-up and statistical analysis

In the first 3 years after radiotherapy, the patients were followed up every 3 months. After 3 years, the follow-up intervals gradually increased from 6 months to 1 year. Follow-up was performed by outpatient review, telephone, or letters. The routine examinations included a complete physical examination, nasopharyngeal endoscopy, blood and biochemistry profiles, chest radiography, abdominal ultrasonography, and CT/magnetic resonance image (MRI) scans of the nasopharynx and cervical region. CT scans of the abdominopelvic cavity or chest, bone scans, and positron emission tomography scans were performed only in symptomatic patients. Patients without recent examination tests in the medical records were followed up by telephone calls. Patients who were lost to follow-up were censored at the last time of contact. Nine patients were lost to follow-up, and the follow-up rate was 80.4%. All endpoints were defined from the starting date of radiotherapy to the date of an event occurrence or the last follow-up.

The statistical analysis was performed using SPSS version 16.0 software (SPSS, Chicago, IL, USA). The actuarial rates were calculated using the Kaplan–Meier method, and the differences were compared using the log-rank test. *P* values less than 0.05 were considered significant.

## Results

### Patient characteristics

The clinical characteristics of the 46 patients are listed in Table 1. Among the patients, 31 were men and 15 were women, with a ratio of 2.07:1. The median age was 43 years (range 26–67 years). Pathologic examinations showed that 43 patients had poorly differentiated squamous cell carcinoma, and three had highly differentiated squamous cell carcinoma.

**Table 1:** Clinical characteristics of the 46 patients with nasopharyngeal carcinoma (NPC)

Characteristic	No. of patients	Percentage (%)
Age (years)		
≤45	26	56.5
>45	20	43.5
Gender		
Male	31	67.4
Female	15	32.6
Clinical stage^a^		
I	3	6.5
II	13	28.3
III	23	50.0
IV	7	15.2
T stage^a^		
T1	8	15.2
T2	9	26.1
T3	22	43.5
T4	7	15.2
N stage^a^		
N0	12	26.1
N1	27	50.0
N2	6	21.7
N3	1	2.2
Histology		
Poorly differentiated	43	93.5
Well differentiated	3	6.5

*T* tumor; *N* node

^a^ According to the 7th American Joint Committee on Cancer (AJCC) staging system for NPC

### Follow-up outcomes

The final follow-up was performed in August 2012, and the median follow-up duration was 99 months (range 9–250 months). A total of 42 patients were followed beyond 10 years. Nine patients were lost to follow-up: three were lost within 3 years after the completion of radiotherapy, and five were lost 10 years after radiotherapy. Of the 46 patients, 42 (91.3%), 42 (91.3%), and 39 (84.8%) had complete follow-up data available at 5, 10, and 20 years, respectively. During follow-up, 30 patients died: 11 died of recurrence, eight died of distant metastasis, and 12 died of other causes (Table 2).

**Table 2:** Causes of death for the 46 NPC patients

Cause	No. of patients	Percentage (%)
Recurrence alone	10	21.7
Nasopharynx	2	4.3
Cervical lymph nodes	4	8.7
Nasopharyngeal and cervical lymph nodes	4	8.7
Distant metastasis alone	7	15.2
Distant metastasis with recurrence in the nasopharynx	1	2.2
Others	12	26.1

### Survival rates

**Fig. 2:**
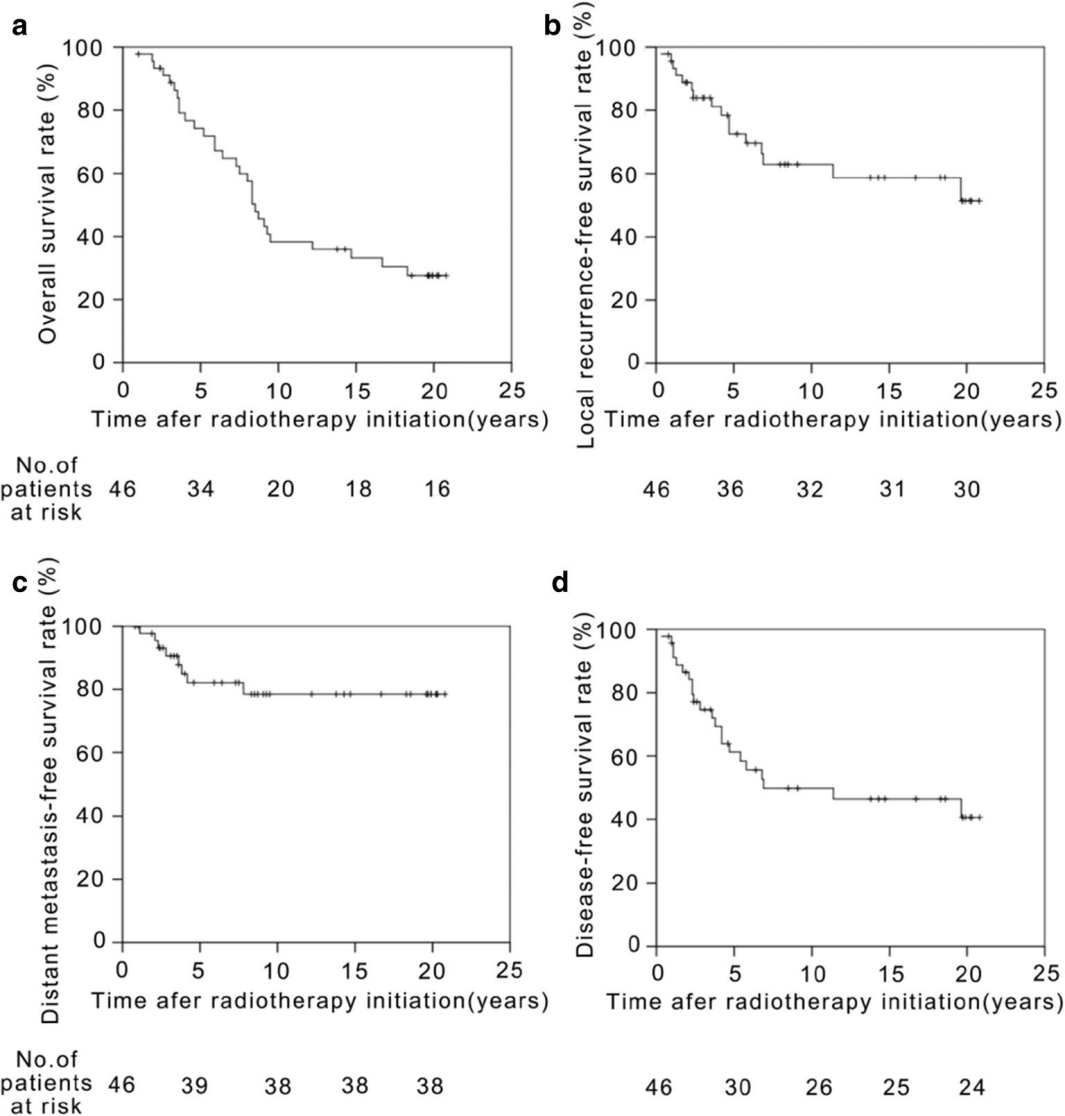
The Kaplan–Meier estimated survival curves of the 46 patients with NPC. **a** overall survival; **b** local recurrence-free survival; **c** distant metastasis-free survival; and **d** disease-free survival

The 5-year OS, local recurrence-free survival (LRFS), distant metastasis-free survival (DMFS), and disease-free survival (DFS) rates were 74.3%, 72.6%, 82.1%, and 61.2%, respectively. The corresponding 10-year rates were 38.4%, 62.9%, 78.5%, and 49.8%, respectively, and the 20-year rates were 27.7%, 51.4%, 78.5%, and 40.7%, respectively (Fig. 2). The 5-, 10-, and 20-year survival rates of patients at different clinical stages, as well as the T and N stages, are summarized in Table 3.

**Table 3:** The 5-, 10-, and 20-year survival rates for the 46 NPC patients at different stages

Item	OS rate (%)			*P* value	LRFS rate (%)			*P* value	DMFS rate (%)			*P* value	DFS rate (%)			*P* value
	5-year	10-year	20-year		5-year	10-year	20-year		5-year	10-year	20-year		5-year	10-year	20-year	
Clinical stage^a^																
I	66.7	66.7	66.7	0.979	66.7	66.7	66.7	0.019	100	100	100	0.072	66.7	66.7	66.7	<0.001
II	70.0	30.0	30.0		92.3	82.1	82.1		82.1	82.1	82.1		75.2	64.5	64.5	
III	73.8	39.1	29.0		73.6	60.2	42.2		90.4	84.0	84.0		70.4	53.2	37.2	
IV	85.7	42.9	14.3		25.7	25.7	25.7		31.2	31.2	31.2		0	0	0	
T stage^a^																
T1	80.0	26.7	26.7	0.996	87.5	58.3	58.3	0.020	85.7	68.6	68.6	0.066	58.3	43.8	43.8	<0.001
T2	66.7	44.4	33.3		88.9	88.9	88.9		88.9	88.9	88.9		77.8	77.8	77.8	
T3	72.7	36.4	30.3		72.1	64.9	45.4		89.9	89.9	89.9		68.6	49.7	39.7	
T4	85.7	42.9	14.3		25.7	25.7	25.7		31.2	31.2	31.2		0	0	0	
N stage^a^																
N0	58.3	29.2	29.2	0.992	80.8	80.8	80.8	<0.001	90.9	90.9	90.9	0.068	71.6	71.6	71.6	0.005
N1	67.9	39.3	31.2		75.6	69.8	55.9		78.3	78.3	78.3		55.4	50.3	40.3	
N2	83.3	50.0	16.7		60.0	20.0	0		80.0	60.0	60.0		60.0	20.0	0	
N3	0	0	0		0	0	0		0	0	0		0	0	0	

*NPC* nasopharyngeal carcinoma; *OS* overall survival; *LRFS* local recurrence-free survival; *DMFS* distant metastasis-free survival; *DFS* disease-free survival

^a^ As defined by the criteria of the seventh edition of the AJCC staging system for NPC

### QoL results

The acute and late radiation toxicity data are reported in Table 4. Most acute toxicities were mild. The major grades 3–4 acute toxicities were mucositis (21.7%) and dermatitis (13.0%).

**Table 4:** Acute and late toxicities in the 46 NPC patients treated with 50 Gy radiotherapy plus hematoporphyrin derivative

Toxicity	Grade 0	Grade 1	Grade 2	Grade 3	Grade 4
Acute toxicities					
Skin (dermatitis)	0	32 (69.6)	8 (17.4)	6 (13.0)	0
Mucositis	0	16 (34.8)	20 (43.5)	10 (21.7)	0
Xerostomia	0	33 (71.7)	13 (28.3)	0	0
Hearing loss	40 (87.0)	6 (13.0)	0	0	0
Late toxicities^a^					
Temporal lobe necrosis	42 (100)	0	0	0	0
Cranial nerve palsy	42 (100)	0	0	0	0
Optic nerve/chiasm injury	42 (100)	0	0	0	0
Brainstem injury	42 (100)	0	0	0	0
Hypopituitarism	42 (100)	0	0	0	0
Hypothyroidism	42 (100)	0	0	0	0
Trismus	35 (83.3)	7 (16.7)	0	0	0
Neck fibrosis	36 (85.7)	6 (14.3)	0	0	0
Xerostomia	3 (7.1)	38 (90.5)	1 (2.4)	0	0
Hearing loss	5 (11.9)	35 (83.3)	2 (4.8)	0	0

All values are presented as the numbers of patients followed by percentages in the parentheses

^a^ The 42 patients who were followed up for at least 10 years are included in the analysis of late toxicities

**Fig. 3:**
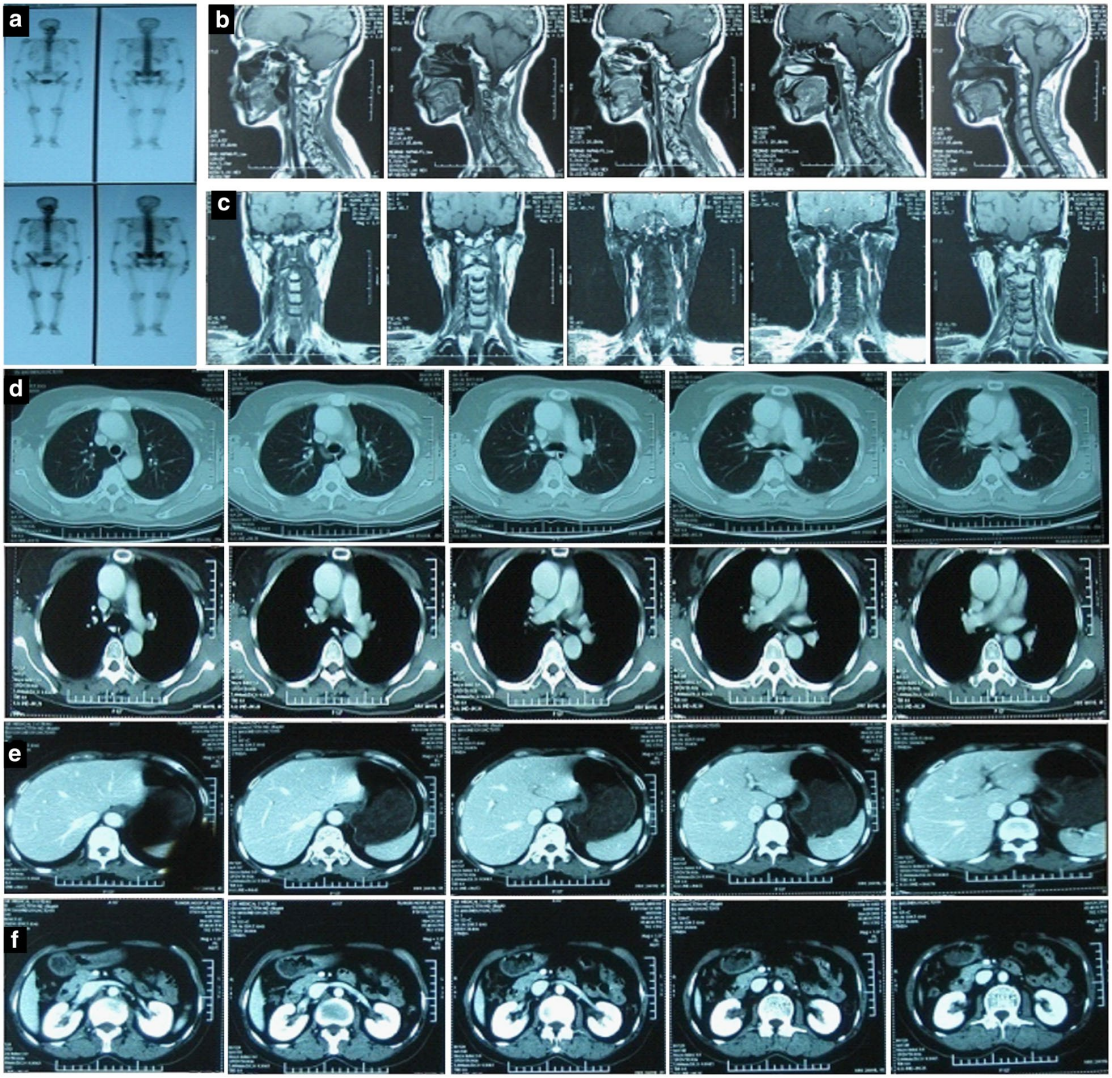
Examination of an NPC patient 20 years after 50 Gy radiotherapy plus HPD. **a** whole body bone scan image without abnormally increased uptake; **b** nasopharyngeal neoplasm disappears on nasopharyngeal magnetic resonance image (MRI); **c** cervical lymph node metastases are not found on cervical MRI; **d** chest computed tomography (CT) is normal; **e** upper abdominal CT without any abnormal lesion; **f** metastases are not found on lower abdominal CT

The occurrence rates of grades 1 and 2 late toxicities were 90.5% and 2.4% for xerostomia and 83.3% and 4.8% for hearing loss, respectively, for the 42 patients who were followed up for at least 10 years. Additionally, of the nine patients who survived for no less than 20 years, none had grade 2 or more severe late complications, two suffered from grade 1 restriction of mouth opening, and six had grade 1 xerostomia and hearing impairment. Temporal lobe necrosis, cranial nerve palsy, optic nerve/chiasm injury, brainstem injury, hypopituitarism, and hypothyroidism were not observed in any patient. Figure 3 shows the whole body bone scan, head and neck MRI, and chest and abdominal CT scan of a NPC patient who survived for 20 years after 50 Gy radiotherapy plus HPD. Her physical and imaging examinations were all normal, and she had a good QoL without any late complications.

## Discussion

In this study, we report on the long-term survival and low occurrence rates of late toxicities for some radiosensitive NPC patients after they received 50 Gy radiotherapy plus HPD. In our study, for the 46 NPC patients who were treated with 50 Gy radiotherapy plus HPD 20 years ago, the 5-, 10- and 20-year DFS rates were 61.2%, 49.8%, and 40.7%, respectively. Our results were close to those reported by Yi et al. [1], who analyzed patients treated with 70–72 Gy between 1990 and 1999 with 5- and 10-year DFS rates of 58.4% and 52.1%, respectively. Because of the relatively small sample size in our study and heterogeneity of the involved population, the survival rates in our study may not be representative, but we have at least confirmed that some radiosensitive NPC patients can be treated with 50 Gy radiation. The 46 NPC patients received HPD during the course of radiotherapy to enhance radiosensitivity. However, recent studies have shown that HPD can’t function as a radiosensitizer for the radiation treatment of cancer [15, 16]. Therefore, it cannot affect the efficacy of radiotherapy.

It is worth mentioning that none of the NPC patients in our study received chemotherapy. Chemotherapy also plays an important role in improving patients’ survival [23–25], which may explain the relatively low 5-year LRFS rate (72.6%) in our study. Additionally, our study is based on a two-dimensional technique. For two-dimensional radiotherapy, the target volume coverage and normal tissue sparing are not well balanced. By contrast, intensity-modulated radiation therapy (IMRT) offers excellent target volume coverage and protects the normal tissue adjacent to the target [26]. Several studies have reported that the use of IMRT had improved the 5-year LRFS rate of patients with non-metastatic NPC from 84% to 93% and had reduced late toxicity as compared with conventional radiotherapy [27, 28]. Thus, if we use chemotherapy combined with low-dose IMRT to treat radiosensitive NPC patients, we can significantly improve both the survival rate and QoL, making the prospect of low-dose radiotherapy for radiosensitive NPC more promising.

Radiotherapy has an important role in treating NPC, but there is major controversy in high-dose conventional radiotherapy concerning severe late toxicities and high incidence of radiation-related toxicities. The radiation-induced complications affect the QoL of NPC patients, especially for pediatric and adolescent NPC patients. Using radiation at doses between 66 and 80 Gy, many researchers have reported occurrence rates of 42.3%–98% for xerostomia [2, 9, 29, 30], 5%–66% for restriction of mouth opening [2, 7, 9, 30, 31], 4.5%–58% for neck fibrosis [2, 7, 9, 29, 30], and 1%–6% for radiation-induced brain necrosis [2, 6, 32]. In our study, there was a moderate incidence of acute toxicities; all patients demonstrated good compliance and successfully completed radiation on the basis of supportive treatment. Of the patients who survived no less than 20 years after receiving 50 Gy radiotherapy, severe radiation-related complications were rarely observed. Six patients (66.7%) developed grade 1 xerostomia and ototoxicity, 2 (22.2%) developed grade 1 restriction of mouth opening, and none developed grade 3–4 toxicities. All survival patients had a good QoL. The rate of radiation-induced complications was lower in patients who received 50 Gy radiotherapy than in those who received high-dose radiation. One possible explanation is that the normal tissue adjacent to the target received a lower radiation dose in the 50 Gy group. Furthermore, these patients may have better tolerance of radiation toxicities as they lived longer and repaired normal tissue. Therefore, compared with conventional radiotherapy, 50 Gy radiotherapy has a distinct advantage in improving the QoL of NPC patients.

A major challenge that we face is to identify radiosensitive NPC patients. Many studies have reported predictive markers for radiosensitivity [33–39]. We also have proposed some markers for identifying radiosensitive NPC, such as DNA-dependent protein kinase catalytic subunit (DNA-PKcs) protein and breast cancer susceptibility gene (BRCA) 1/BRCA2-containing complex, subunit three (BRCC3) [40, 41]. However, the detection of these sophisticated indictors is difficult in practice due to the restrictions in the current diagnostic and treatment techniques. Hence, a radiosensitivity categorization system with a delicate balance of accuracy and practicality should be considered. Additionally, further studies are needed to identify the target patients and provide individualized radiation treatment.

Our study has several limitations. First, 20 years is a long follow-up time; because of the changes in the residential addresses and contact information, the rate of follow-up is not sufficiently high and our findings are incomplete. Second, the cohort was selected from a specific, regionally based population that may not be representative of the general population of NPC patients. Third, the limited sample size and low numbers of positive events in our study could be a potential source of bias. Fourth, 85% of the patients had stages I-III NPC in this study, which may be explained by that small tumors do not require high radiation doses for tumor control because of the log-cell-kill principle of radiation treatment. However, we failed to compare small versus large tumors in our analysis due to the small sample size. The association of clinical stage with the radiosensitivity of NPC should be further assessed. Fifth, the patients were treated with radiotherapy plus HPD. Although HPD doesn’t affect radiosensitivity, it has exhibited an antiproliferative effect on various human cancer cells in photodynamic therapy [42, 43], and its role in the treatment of NPC requires further investigation. Finally, identifying radiosensitive NPC patients is still a problem that requires further investigation, and we should be cautious about using low-dose radiotherapy to treat NPC. For these reasons, we must validate our findings in a larger sample of patients with a multi-institutional prospective study design.

## Conclusion

In a subset of radiosensitive NPC patients, 50 Gy radiotherapy conferred a high long-term survival rate and good QoL.
